# Forced Exercise Preconditioning Attenuates Experimental Autoimmune Neuritis by Altering T_h_1 Lymphocyte Composition and Egress

**DOI:** 10.1177/1759091415595726

**Published:** 2015-07-16

**Authors:** Michael W. Calik, Sahadev A. Shankarappa, Kelly A. Langert, Evan B. Stubbs

**Affiliations:** 1 Center for Narcolepsy, Sleep and Health Research, Department of Biobehavioral Health Science, University of Illinois at Chicago, Chicago, IL, USA; 2 Department of Veterans Affairs, Edward Hines Jr. VA Hospital, Hines, IL, USA; 3 Program in Neuroscience, Stritch School of Medicine, Loyola University Chicago, Maywood, IL, USA; 4 Center for Nanoscience and Molecular Medicine, Amrita institute of Medical Sciences, Amrita Vishwa Vidyapeetham, Kochi, Kerala, India; 5 Department of Ophthalmology, Stritch School of Medicine, Loyola University Chicago, Maywood, IL, USA

**Keywords:** exercise, preconditioning, peripheral nerve, experimental autoimmune neuritis, Guillain–Barré syndrome

## Abstract

A short-term exposure to moderately intense physical exercise affords a novel measure of protection against autoimmune-mediated peripheral nerve injury. Here, we investigated the mechanism by which forced exercise attenuates the development and progression of experimental autoimmune neuritis (EAN), an established animal model of Guillain–Barré syndrome. Adult male Lewis rats remained sedentary (control) or were preconditioned with forced exercise (1.2 km/day × 3 weeks) prior to P2-antigen induction of EAN. Sedentary rats developed a monophasic course of EAN beginning on postimmunization day 12.3 ± 0.2 and reaching peak severity on day 17.0 ± 0.3 (*N* = 12). By comparison, forced-exercise preconditioned rats exhibited a similar monophasic course but with significant (*p* < .05) reduction of disease severity. Analysis of popliteal lymph nodes revealed a protective effect of exercise preconditioning on leukocyte composition and egress. Compared with sedentary controls, forced exercise preconditioning promoted a sustained twofold retention of P2-antigen responsive leukocytes. The percentage distribution of pro-inflammatory (T_h_1) lymphocytes retained in the nodes from sedentary EAN rats (5.1 ± 0.9%) was significantly greater than that present in nodes from forced-exercise preconditioned EAN rats (2.9 ± 0.6%) or from adjuvant controls (2.0 ± 0.3%). In contrast, the percentage of anti-inflammatory (T_h_2) lymphocytes (7–10%) and that of cytotoxic T lymphocytes (∼20%) remained unaltered by forced exercise preconditioning. These data do not support an exercise-inducible shift in T_h_1:T_h_2 cell bias. Rather, preconditioning with forced exercise elicits a sustained attenuation of EAN severity, in part, by altering the composition and egress of autoreactive proinflammatory (T_h_1) lymphocytes from draining lymph nodes.

## Introduction

Visceral-type obesity, secondary to a sedentary lifestyle, is a marked independent risk factor for the development and progression of several subacute or chronic diseases (Rivera-Vega et al., 2014; Versini et al., 2014; Buryk et al., 2015; Procaccini et al., 2015). Moderately intense physical exercise, performed at regular intervals, is now firmly recognized as a safe and effective intervention by which to enhance quality of life, largely secondary to improved mental and physical health (Apostolopoulos et al., 2014). The physiologic response to exercise is multifactorial, affecting musculoskeletal, cardiovascular, respiratory, endocrine, neural, and humoral and adaptive immune systems. Early studies report that moderate exercise enhances immune system function (Pedersen and Ullum, 1994; Shephard et al., 1995). By altering immune-mediated responses, exercise is emerging as a viable adjunctive therapeutic strategy for the management of disorders as seemingly diverse as anxiety, depression, cancer, cardiovascular disease, metabolic syndrome, Type 2 diabetes, and autoimmunity (McFarlin et al., 2006; Ploeger et al., 2009; Speck et al., 2010; Gleeson et al., 2011; Gordon et al., 2012; Apostolopoulos et al., 2014).

The mechanisms by which exercise alters innate or adaptive immunity in the normal, sedentary (nonathletic) population remain enigmatic and are critically dependent on the type, intensity, and duration of physical exercise administered (Nieman, 1995; Nieman et al., 1995a; Nieman et al., 1995b; Nielsen, 2003). In general, acute exercise is frequently associated with enhanced proinflammatory signaling, as measured by induction of macrophages (M1), CD4^+^ T cells (T_h_1), and CD8^+^ cytotoxic T cells. Paradoxically, acute aerobic or resistance training exercise programs, however, are reported to significantly *decrease* the risk and severity of some inflammatory autoimmune diseases (Courtney et al., 2011; Golbidi et al., 2012; Perandini et al., 2012). In some subjects, intense chronic physical exercise may actually lead to a collapse of natural immunity (Cook et al., 2013; Thomas, 2013).

The underlying cellular mechanisms by which physical exercise may modulate immune responses remain poorly understood but may be attributed to changes in the functional status (T_h_1 vs. T_h_2; M1 vs. M2) of inflammatory immune cells (Pedersen, 2011; Goh et al., 2014). Proposed as a novel source of anti-inflammatory cytokines (Pedersen, 2011), repeated bouts of contracting skeletal muscle may shift the balance of circulating monocyte or T-cell populations from a pro-inflammatory (M1, T_h_1) to that of an anti-inflammatory (M2, T_h_2) profile. By comparison, peripheral blood from endurance-trained athletes contains elevated levels of interleukin-10 (IL-10) and a greater population of CD4^+^CD25^+^CD127^low^ T regulatory cells, both anti-inflammatory markers (Handzlik et al., 2013). Alternatively, a 30-min session of aerobic cycling is reported to upregulate expression of both pro- (tumor necrosis factor-α [TNF-α] and IL-6) and anti-inflammatory (IL-4) cytokines, suggesting that acute aerobic exercise may precondition (or prime) T_h_1/M1 and T_h_2/M2 immune mediators (Zaldivar et al., 2006).

Experimentally, forced exercise has been shown to attenuate development and progression of humoral and/or cellular autoimmunity in animal models of rheumatoid arthritis (Navarro et al., 2010), multiple sclerosis (Bernardes et al., 2013), Guillain–Barré syndrome (Calik et al., 2012), as well as significantly delaying the development of lung damage (Hung et al., 2013) and neuropathic pain (Shankarappa et al., 2011; Chen et al., 2013) in the streptozotocin (STZ)-diabetic animal model. Here, rats preconditioned with forced exercise were found to exhibit a sustained protection against the development and progression of experimental autoimmune neuritis (EAN), an established CD4^+^ T-cell dependent rat model of human inflammatory demyelinating neuropathies. The protective effect of preconditioning was not due to a shift in the T_h_1:T_h_2 cell bias but rather appears to be the result of altering autoreactive leukocytes composition and egress from secondary lymphoid tissue.

## Methods and Materials

This study was conducted using protocols approved by the Institutional Animal Care and Use Committee in accordance with the principles of laboratory animal care (NIH publication No. 86-23, 1985). Animals were housed three to a cage, allowed standard rat chow and water *ad libitum,* and maintained on a 12 h/12 h light/dark cycle. Adult male Lewis rats (initial body weight 200 g; Harlan, Indianapolis, IN, USA) were randomly divided into adjuvant control, sedentary, or forced-exercise intervention groups.

### Forced Exercise Preconditioning

Treadmill running is a well-established forced experimental training method that elicits marked adaptations in rodents. Rats assigned to the forced-exercise preconditioned intervention group were acclimated (5-day training period) to a motorized treadmill (Exer 3/6 Open treadmill, Columbus Instruments, Columbus, OH). Although equipped with a motivational shock grid, this treadmill feature was not used in an effort to minimize stress-induced physiological changes. Training involved a gradual transition at a zero grade incline toward a constant velocity (20 m/min) and duration (60 min/day; 1.2 km/day/rat) as previously described (Shankarappa et al., 2011; Calik et al., 2012). Rats acclimated to treadmill training were run for 60 min/day × 5 days a week between the hours of 10:00 h to 13:00 h for an additional 3 weeks. Rats assigned to the sedentary intervention group were allowed to explore an identically sized environment for the same duration of time during the same time of day, but without receiving an exercise challenge. Rats assigned to the adjuvant control group were housed in their home cages and received the same amount of handling. All rats were weighed daily, and caloric balance between adjuvant, sedentary, and forced-exercise preconditioned rats was not monitored. Although all three groups of rats steadily gained body weight throughout this study, rats undergoing forced exercise gained weight at a reduced rate compared with sedentary control rats (Calik et al., 2012). At no time prior to EAN induction, however, did forced-exercise preconditioned rats exhibit a frank loss of body weight. Relative changes in muscle mass or adrenal gland weights were not determined.

### EAN Induction

Following a 3-week training regimen, sedentary and forced-exercise preconditioned rats were actively induced with EAN as previously described (Sarkey et al., 2007; Calik et al., 2012). Rats were anesthetized with ketamine (90 mg/kg)-xylazine (7.5 mg/kg) and 100 µl of a freshly prepared fine-particle emulsion (1:1 v/v) containing 100 µg of a synthetic neuritogenic P2 peptide (53–78, Dana-Farber Cancer Institute, Harvard University, Boston, MA, USA) suspended in sterile saline and incomplete Freund’s adjuvant (Sigma–Aldrich) supplemented with 10 mg/ml heat-inactivated *Mycobacteria tuberculosis* (strain H37Ra; Difco Laboratories, Detroit, MI, USA) was injected into the left hind footpad. Inactive adjuvant control rats were injected in the same way except neuritogenic P2 peptide was omitted.

All rats were scored daily for EAN development and progression by investigators blinded to group assignment. The severity of clinical signs was scored as follows: 0 = *no symptoms*; 1 = *flaccid tail*; 2 = *abnormal gait*; 3 = *mild paraparesis*; 4 = *severe paraparesis*; 5 = *paraplegia*. Intermediate clinical signs were scored in increments of 0.5.

At peak of disease, sciatic nerves from sedentary or forced-exercise preconditioned rats were harvested and prepared for qualitative histopathological evaluation as previously described (Sarkey et al., 2007).

### Leukocyte Function Studies

P2-antigen-specific proliferation and cytokine production assays were performed as we have previously described (Sarkey et al., 2007). Adjuvant control, sedentary, or forced-exercise preconditioned EAN rats were sacrificed by CO_2_ asphyxiation at either onset (Day 14) or peak (Day 18) of disease, and secondary lymphoid tissue (spleens and popliteal lymph nodes) was harvested. Prepared splenic or nodal lymphocytes were cultured at an initial density of 2.0 × 10^6^ cells/ml in Roswell Park Memorial Institute-1640 media supplemented with 9% heat-inactivated fetal bovine serum, 0.05 μM 2-mercaptoethenol, 90 U/ml penicillin, and 90 µg/ml streptomycin. Cells were cultured in the absence or presence of P2 antigen (10 µg/ml) for 96 h. For the final 24 h of antigen stimulation, 0.5 µCi of [^3^H]thymidine (6.7 Ci/mmol; MP Biomedicals, USA) was added to each well. Labeled cells were harvested by trypsinization and [^3^H]thymidine incorporation into the splenic or nodal lymphocyte DNA was determined using a Tri-Carb 2810 liquid scintillation analyzer (Perkin Elmer, USA). Cell culture supernatants from parallel nonradiolabeled assays were collected at 96 h and analyzed for the presence of interferon-γ (IFN-γ), TNF-α, IL-2, IL-6, and IL-10. The content of cytokines released into culture media was quantified simultaneously using multiplex technology with the Bio-plex™ system (Bio-Rad Laboratories, Inc., Hercules, CA, USA) and a commercially available rat cytokine Milliplex kit (Millipore, USA).

### Flow Cytometry

Spleens or popliteal lymph nodes were harvested, and trunk blood was collected (in 5 mM EDTA), at onset (Day 14) or peak (Day 18) of disease from adjuvant control, sedentary, or forced-exercise preconditioned EAN rats. To determine the relative (percent) abundance of helper (T_h_) and cytotoxic (T_c_) T lymphocytes retained in these secondary lymphoid tissues, 1 × 10^6^ splenocytes, nodal, or circulating leukocytes were co-incubated for 30 min at 4℃ with 1 µg each of fluorescein isothiocyanate (FITC)-conjugated mouse anti-rat CD4 (clone W3/25) and phycoerythrin (PE)-conjugated mouse anti-rat CD8α (clone G28) monoclonal antibodies (BioLegend, USA).

The abundance of T_h_1 lymphocytes or activated T_c_ lymphocytes was determined by co-incubating prepared splenocytes, nodal, or circulating leukocytes with 1 µg each of FITC-conjugated mouse anti-rat CD4 (clone W3/25) or PE-conjugated mouse anti-rat CD8α (clone G28) monoclonal antibody together with 1 µg of rabbit anti-rat IL-12Rβ1 (CD212, clone C-20) followed by 1 µg of allophycocyanin (APC)-conjugated goat anti-rabbit IgG (H + L) secondary antibody.

The abundance of T_h_2 lymphocytes was determined by co-incubating prepared splenocytes, nodal, or circulating leukocytes with 1 µg each of FITC-conjugated mouse anti-rat CD4 (clone W3/25) and PE-conjugated hamster anti-rat CD278 (clone 398.4 A) monoclonal antibodies.

Co-immunostained lymphocytes were washed twice with ice-cold bovine calf serum (5% in phosphate buffer saline) and resuspended in 1 ml of ice-cold buffered (pH 7.4) 4% paraformaldehyde. After 10 min, fixed cells were washed twice and resuspended in serum-supplemented phosphate buffer saline. Data were collected using a fluorescence-activated cell sorting (FACS) Canto flow cytometer (BD Biosciences, San Jose, CA, USA) and analyzed with FlowJo software (Tree Star, Inc., USA). Lymphocytes were gated according to their forward and side scatter. In all cases, isotype controls were used to quantify and correct for background fluorescence.

### Statistical Analysis

Data are expressed as mean ± SEM of *N* observations unless otherwise specified. Statistical significance between nonparametric clinical data was determined using a Mann–Whitney nonparametric *U* test. Analysis of multiple variable parametric data was determined using either one-way analysis of variance (ANOVA) or two-way repeated measures ANOVA with Tukey’s or Bonferonni’s post hoc analysis, as indicated. In all cases, *p* < .05 was considered statistically significant.

## Results

### Forced Exercise Preconditioning Attenuates the Severity of EAN

**Figure 1.**
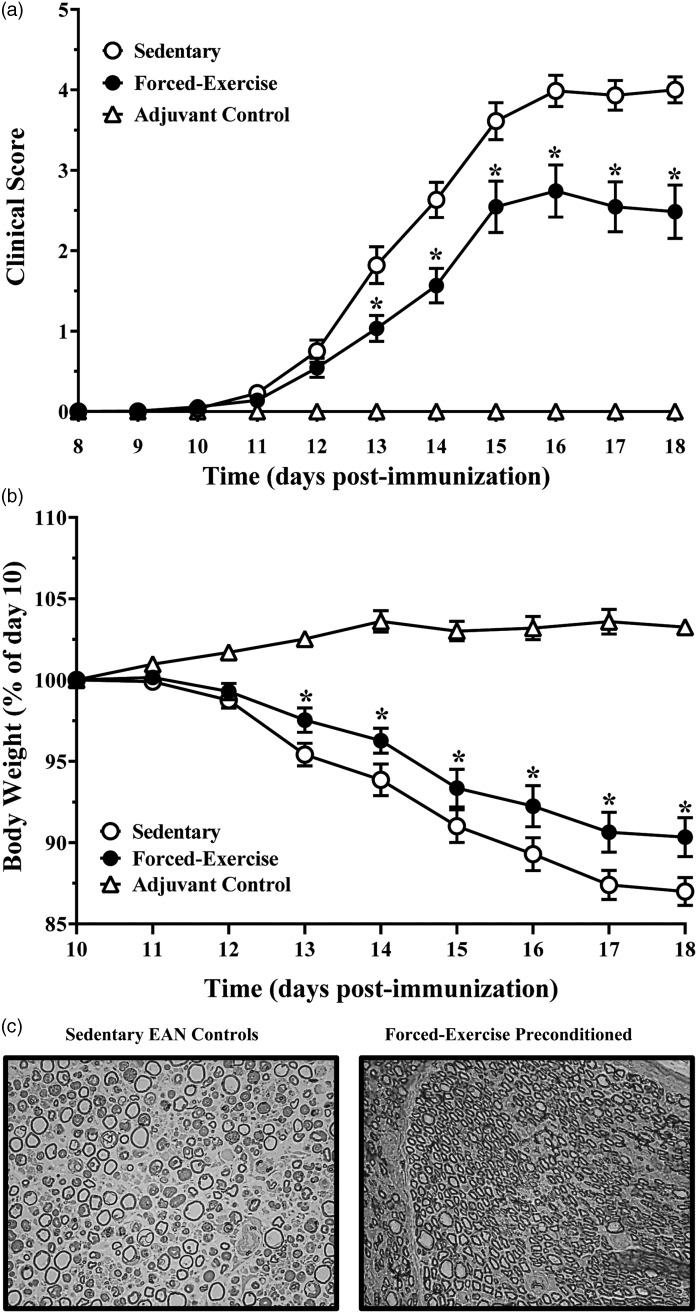
Forced exercise preconditioning attenuates development and progression of EAN. Relative changes in (a) clinical development and progression of EAN and of (b) body weights of adolescent male Lewis rats immunized without (adjuvant control) or with synthetic P2-antigenic peptide following 3 weeks of sedentary or forced exercise preconditioning, as indicated. Data shown are the means ± SEM (*N* = 5–24). *p < .05, forced exercise vs. sedentary control determined by (a) Mann–Whitney nonparametric *U* test or (b) repeated measures ANOVA with Tukey’s post hoc analysis. (c) Histological light micrographic transverse sectional (0.5 µm thick) images of osmicated Embed-812 embedded toluidine blue stained sciatic nerves harvested at peak of disease from sedentary (left panel) or forced-exercise preconditioned (right panel) EAN rats. Images are representative of five to six rats per group. EAN = experimental autoimmune neuritis; ANOVA = analysis of variance.

Following a 3-week regimen of sedentary- or forced exercise preconditioning, P2-immunized rats exhibited a monophasic course of moderate to severe paraparesis that is characteristic of EAN (Sarkey et al., 2007; Calik et al., 2012). Sedentary rats exhibited onset of EAN beginning on postimmunization day 12.3 ± 0.2 (*N* = 24), with peaked severity observed at day 17.0 ± 0.3 (*N* = 12). By comparison, forced-exercise preconditioned rats exhibited a similar monophasic disease course, with onset beginning on postimmunization day 12.6 ± 0.2 (*N* = 21) and peaking on day 15.7 ± 0.2 (*N* = 10). Although forced exercise preconditioning did not significantly alter the monophasic course of EAN, disease severity in these rats was significantly less than that observed in sedentary EAN rats (Figure 1(a)), consistent with our previously reported findings (Calik et al., 2012). In contrast, complete Freund's adjuvant (CFA) only-immunized control rats (adjuvant control animals) did not develop EAN (Figure 1(a)).

Despite having access to water and food *ad libitum*, sedentary EAN rats lost approximately 15% of their postimmunization day-10 body weight, consistent with this well-established measure of onset and progression of EAN. Similarly, the body weights of forced-exercise preconditioned EAN rats also decreased with disease progression, albeit to a significantly lesser degree (Figure 1(b)). Adjuvant control rats, by comparison, did not exhibit weight loss during the course of the study (Figure 1(b)).

In good agreement with our previous reported findings (Sarkey et al., 2007), sciatic nerves harvested at peak of disease from sedentary EAN rats exhibited marked histopathological evidence of edema, inflammatory infiltrates, myelin ovoids, and axonal damage (Figure 1(c), left panel). In contrast, forced-exercise preconditioned EAN rats exhibited qualitatively less histopathological evidence of peripheral nerve injury (Figure 1(c), right panel).

### Forced Exercise Preconditioning Promotes Retention of Leukocytes in Popliteal Lymph Nodes

**Figure 2.**
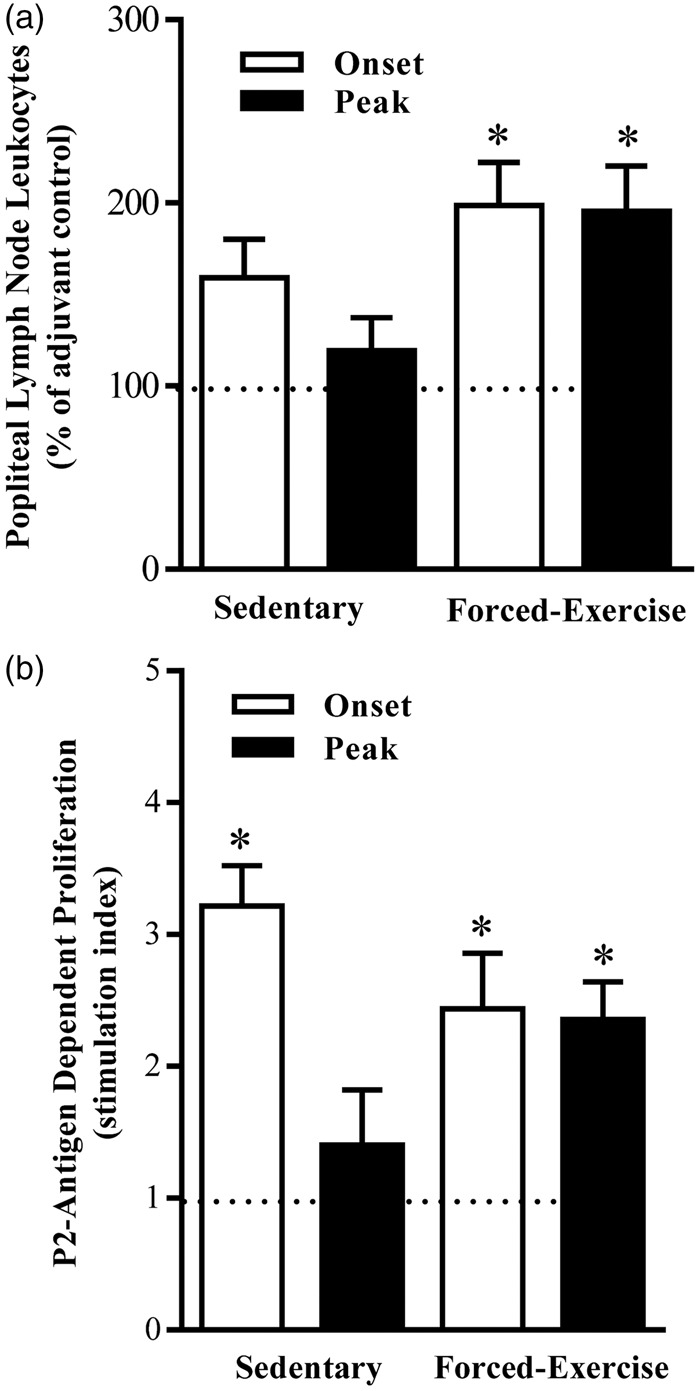
Forced exercise preconditioning promotes nodal retention of P2-antigen-responsive leukocytes. (a) Total number and (b) P2-antigen-induced proliferation of popliteal lymph node leukocytes harvested at onset or peak of disease from adjuvant control (dashed line), sedentary, or forced-exercise preconditioned EAN rats. Data shown are the means ± SEM (*N* = 5–19). *p < .05 versus adjuvant control*,* one-way ANOVA with Bonferroni’s post hoc analysis. EAN = experimental autoimmune neuritis; ANOVA = analysis of variance.

To examine whether forced exercise preconditioning attenuates EAN severity by altering nodal egress of autoreactive lymphocytes, popliteal lymph nodes from sedentary or forced-exercise preconditioned EAN rats were harvested and retention of total and P2-responsive leukocytes quantified (Figure 2). Although the total number of leukocytes retained in popliteal nodes from sedentary EAN rats was not significantly different from adjuvant controls, forced-exercise preconditioned EAN rats exhibited a marked twofold increase in total nodal cells recovered at either onset or peak of disease (Figure 2(a)). In contrast, forced exercise preconditioning did not alter the retention of splenic leukocytes (splenocytes, data not shown).

### Forced Exercise Preconditioning Preserves P2-Antigen Responsiveness of Nodal Leukocytes

Nodal leukocytes recovered at disease onset from sedentary EAN rats proliferated robustly to P2-antigen stimulation (Figure 2(b)). In contrast, P2-responsiveness of nodal leukocytes harvested at peak of disease from sedentary EAN rats was significantly (*p* < .05) blunted (Figure 2(b)). By comparison, nodal leukocytes harvested from forced-exercise preconditioned EAN rats at either onset or peak of disease retained their proliferative responsiveness to P2 antigen (Figure 2(b)).

Nodal leukocytes harvested at disease onset from exercise-preconditioned rats exhibited constitutive IL-2 release (98 ± 30 pg/ml, *N* = 5) that was similar to leukocytes from sedentary (79 ± 20 pg/ml, *N* = 5) or adjuvant (85 ± 36 pg/ml, *N* = 5) control rats. By comparison, constitutive release of IL-2 from nodal leukocytes harvested from sedentary or adjuvant control rats at peak of disease was significantly less (31 ± 17 pg/ml, *N* = 5; *p* < .05). In contrast, nodal leukocytes harvested from exercise-preconditioned rats at peak of disease retained their ability to constitutively release IL-2 (109 ± 18 pg/ml, *N* = 5; *p* < .05).

**Figure 3.**
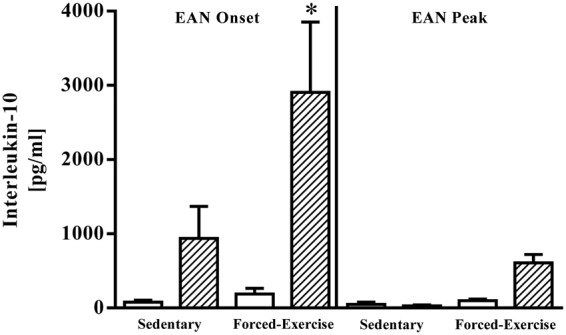
Forced exercise preconditioning enhances nodal leukocyte P2-antigen-stimulated production of anti-inflammatory IL-10. Comparative changes in interleukin-10 present in culture media of vehicle- (open bars) or P2-antigen-stimulated (hatched bars) nodal leukocytes harvested at onset or peak of disease from sedentary or forced-exercise preconditioned EAN rats. Data shown are the means ± SEM (*N* = 4–5). *p < .05 versus paired vehicle-treated leukocytes*,* one-way ANOVA with Bonferroni’s post hoc analysis. EAN = experimental autoimmune neuritis; IL-10 = interleukin-10; ANOVA = analysis of variance.

**Figure 4.**
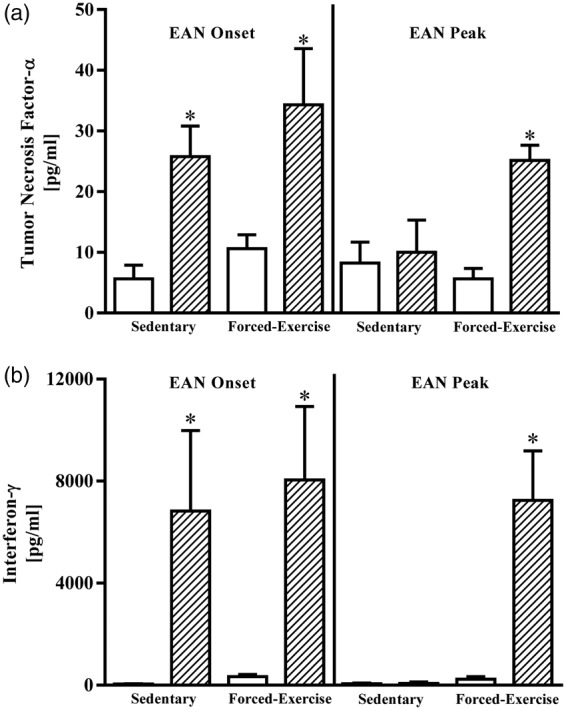
Forced exercise preconditioning preserves nodal leukocyte P2-antigen-stimulated production of pro-inflammatory cytokines. Comparative changes in (a) tumor necrosis factor-α or (b) interferon-γ present in culture media of vehicle- (open bars) or P2-antigen-stimulated (hatched bars) nodal leukocytes harvested at onset or peak of disease from sedentary or forced-exercise preconditioned EAN rats. Data shown are the means ± SEM (*N* = 4–5). *p < .05 versus paired vehicle-treated leukocytes*,* one-way ANOVA with Bonferroni’s post hoc analysis. EAN = experimental autoimmune neuritis; ANOVA = analysis of variance.

To determine whether retained nodal leukocytes were functionally competent, we quantified constitutive and P2-antigen-stimulated production of anti- (Figure 3) and pro-inflammatory cytokines (Figure 4). Challenging nodal leukocytes with P2 antigen elicited marked changes in secreted IL-6, IL-10, TNF-α, and IFN-γ. Compared with vehicle-stimulated controls (17 ± 2 pg/ml, *N* = 5), nodal leukocytes harvested from sedentary rats at disease onset exhibited a twofold increase in P2-antigen-stimulated IL-6 release (36 ± 15 pg/ml, *N* = 5; *p* < .05). By comparison, exercise preconditioning did not alter P2-antigen-stimulated IL-6 release (40 ± 10 pg/ml, *N* = 5). At peak of disease, P2-antigen-stimulated release of IL-6 from nodal leukocytes was statistically indistinguishable from vehicle-stimulated controls (data not shown).

Challenging nodal leukocytes harvested from sedentary rats at disease onset with P2 antigen produced a measurable increase in IL-10 release that was markedly potentiated by exercise preconditioning (Figure 3). At peak of disease, however, P2-antigen-stimulated release of IL-10 was statistically indistinguishable from vehicle-stimulated controls (Figure 3).

P2-antigen also elicited significant increases in TNF-α and IFN-γ production from nodal leukocytes (Figure 4). At onset of disease, nodal leukocyte TNF-α and IFN-γ cytokine responsiveness to P2-antigen was not altered by forced exercise preconditioning (Figure 4). At peak of disease, however, nodal leukocytes harvested from sedentary rats were refractive to P2-antigen stimulation. In marked contrast, nodal leukocytes harvested from forced-exercise preconditioned rats at peak of disease retained their P2-antigen responsiveness (Figure 4(a) and (b)).

### Forced Exercise Preconditioning Does Not Alter T_h_1:T_h_2 Bias in EAN

The ratio of T_h_1:T_h_2 CD4^+^ lymphocytes retained in lymphoid tissue harvested from sedentary or forced-exercise preconditioned EAN rats was determined by flow cytometric analysis. Although forced exercise preconditioning elicited an increase in total leukocyte retention in popliteal lymph nodes (Figure 2), the percentage distribution of single-gated CD4^+^ (T_h_) or CD8^+^ (T_c_) lymphocytes recovered from the popliteal lymph nodes of forced-exercise preconditioned EAN rats was statistically indistinguishable from the nodes of sedentary EAN rats or adjuvant controls (Tables 1 and 2). Similarly, the percentage of CD4^+^ or CD8^+^ lymphocytes recovered from spleens of sedentary (T_h_: 49.6 ± 1.3%, *N = *9; T_c_: 15.3 ± 2.1%, *N = *6) or forced-exercise preconditioned (T_h_: 46.4 ± 1.2%, *N = *9; T_c_: 17.3 ± 1.1%, *N = *6) EAN rats was also statistically indistinguishable.

**Table 1.** Percent Distribution of T Lymphocytes in Popliteal Lymph Nodes Harvested at Onset of EAN.

	T_h_ (CD4^+^)	T_h_1 (CD4^+^CD212^+^)	T_h_2 (CD4^+^CD278^+^)	Tc (CD8^+^)	Tc active (CD8^+^ CD212^+^)
Adjuvant control	56.1 ± 1.1	2.0 ± 0.3	8.7 ± 0.5	18.5 ± 2.4	3.1 ± 0.7
Sedentary EAN	59.1 ± 1.8	5.1 ± 0.9*	7.0 ± 0.9	20.5 ± 1.3	3.3 ± 0.3
Forced-Exercise EAN	55.1 ± 1.8	2.9 ± 0.6	7.5 ± 0.6	21.9 ± 2.4	3.5 ± 0.4

* *Note*. EAN = experimental autoimmune neuritis; ANOVA = analysis of variance. Two-way ANOVA with Bonferroni’s multiple comparison post hoc analysis of (N = 5–8) observations. *p* < .05

**Table 2.** Percent Distribution of T Lymphocytes in Popliteal Lymph Nodes Harvested at Peak of EAN.

	T_h_ (CD4^+^)	T_h_1 (CD4^+^CD212^+^)	T_h_2 (CD4^+^CD278^+^)	T_c_ (CD8^+^)	T_c_ active (CD8^+^ CD212^+^)
Adjuvant control	54.7 ± 1.7	2.2 ± 0.4	7.5 ± 0.9	21.2 ± 1.8	3.6 ± 0.9
Sedentary EAN	59.1 ± 2.7	4.1 ± 0.7	8.1 ± 0.7	19.3 ± 1.4	3.5 ± 1.0
Forced-Exercise EAN	56.0 ± 1.2	3.1 ± 0.3	10.0 ± 0.8	19.3 ± 1.3	2.7 ± 0.2

*Note*. EAN = experimental autoimmune neuritis; ANOVA = analysis of variance. Two-way ANOVA with Bonferroni’s multiple comparison post hoc analysis of (*N* = 5–8) observations.

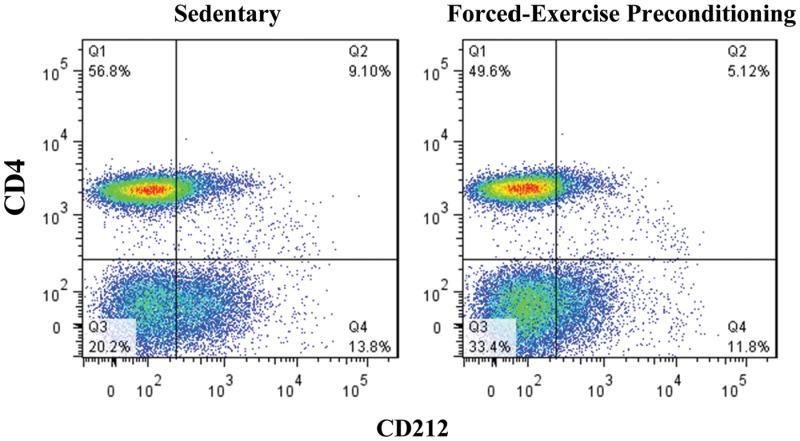

Representative flow cytometric distribution dot plots of nodal T lymphocytes. Lymphocytes present in popliteal lymph nodes harvested at onset of disease from sedentary or forced-exercise preconditioned EAN rats were selected by gating according to their forward and side scatter and T_h_1 subsets subsequently quantified by co-staining with fluorescently tagged monoclonal antibodies to cell surface markers CD4 and CD212, as indicated. In all cases, isotype controls were used to quantify and correct for background fluorescence.

By comparison, the percentage distribution of single-gated CD4^+^ lymphocytes circulating in the blood of sedentary EAN rats (56.0 ± 1.0%, *N = *6) at peak of disease was similar to that of adjuvant controls (58.2 ± 0.6%, *N = *3). Forced exercise preconditioning did not significantly alter the distribution of circulating CD4^+^ lymphocytes (57.5 ± 2.4%, *N = *5) or alter the distribution of circulating cytotoxic T lymphocytes (20.9 ± 0.6%, *N = *5 *vs.* 23.0 ± 1.1%, *N = *6 sedentary EAN rats). Subset analysis of CD4^+^ lymphocytes showed, however, that at peak of disease, sedentary EAN rats exhibited a modest increase in circulating CD4^+^ CD212^+^ (T_h_1) lymphocytes (6.2 ± 0.9%, *N = *6) compared with adjuvant controls (4.0 ± 0.9%, *N = *3). In contrast, the distribution of circulating T_h_1 lymphocytes from forced-exercise preconditioned rats was similar (3.6 ± 0.8%, *N = *5) to that of adjuvant controls. The distribution of circulating CD4^+^ CD278^+^ (T_h_2) lymphocytes (7.2 ± 0.3%, *N = *6) was, however, not significantly altered by forced exercise preconditioning (7.6 ± 1.3%, *N = *5), arguing against an exercise-inducible shift in T_h_1:T_h_2 bias.

Subset analysis of CD4^+^ lymphocytes in popliteal lymph nodes harvested at disease onset from sedentary EAN rats revealed an approximate 2.5-fold increase in retained T_h_1 lymphocytes percentage distribution compared with nodes harvested from adjuvant control rats (Table 1). In contrast, the distribution of T_h_1 lymphocytes present in nodes harvested at disease onset from forced-exercise preconditioned rats was similar to that of adjuvant controls (Table 1). A qualitatively similar effect of forced exercise preconditioning was seen with T_h_1 lymphocytes recovered from nodes at peak of disease (Table 2) or from spleen (data not shown). The distribution of T_h_2 lymphocytes (7–10%) and that of cytotoxic T lymphocytes (∼20%) present in nodes (Tables 1 and 2) or spleen (data not shown) from EAN rats was similar to that of adjuvant controls and remained unaltered by forced exercise preconditioning.

## Discussion

In this study, we show that preconditioning with forced exercise elicits a novel and sustained attenuation of EAN, in part, by altering the composition and egress of autoreactive pro-inflammatory (T_h_1) lymphocytes from draining popliteal lymph nodes. Compared with sedentary controls, forced exercise preconditioning promoted a sustained twofold retention of P2-antigen-responsive leukocytes within popliteal lymph nodes. The percentage distribution of T_h_1 lymphocytes retained in nodes from sedentary EAN rats was significantly greater than that present in nodes from forced-exercise preconditioned EAN rats or that of adjuvant controls. An exercise-inducible shift in T_h_1:T_h_2 cell bias, however, is not supported as the percentage of anti-inflammatory (T_h_2) lymphocytes and that of cytotoxic T lymphocytes remained unaltered by forced exercise preconditioning.

Previously, we reported that forced exercise (treadmill running, 1.2 km/day × 6 weeks) administered to adult male Lewis rats prior to and during the development of EAN (an established CD4^+^ T-cell dependent rat model of human inflammatory demyelinating neuropathies) affords a novel measure of protection against the development of autoimmune-associated deficits in peripheral nerve function (Calik et al., 2012). The mechanism by which forced exercise protects against EAN-induced peripheral nerve injury appeared unrelated to steroid-induced immune suppression, as total plasma corticosterone content was reduced by 46%, while the levels of circulating corticosteroid binding globulin were elevated. Given the importance of systemic inflammation as a recognized risk factor in several subacute or chronic diseases, including autoimmune disorders, we determined whether forced exercise elicits protection against EAN by altering P2-antigen-induced adaptive autoimmune responses.

A single immunizing dose of CFA-emulsified neuritogenic P2 antigen administered to the footpad of adult male Lewis rats elicits a robust and reproducible immune response that culminates in the development and progression of EAN. This, however, produces localized inflammation that hinders any subsequent physical activity. We therefore designed this study to determine the effect of exercise *preconditioning* on development and progression of *subsequently* induced EAN. Although forced-exercise preconditioning did not alter the course of EAN, disease severity was significantly less than that seen with sedentary controls. While physical exercise is often advocated as a safe and effective adjunctive therapeutic strategy for the management of a variety of seemingly diverse disorders, this is the first report to our knowledge that shows exercise *preconditioning* eliciting a *sustainable* protective effect against the development of EAN, a well-established experimental model of an autoimmune inflammatory neurodegenerative disease.

Earlier studies suggest that physical exercise may protect against subacute or chronic inflammation by actively suppressing pro-inflammatory innate (Lancaster et al., 2005) or adaptive (Flynn et al., 2007; Neto et al., 2011) immune responses. In general, a pro-inflammatory (T_h_1) adaptive immune response is a subsequent acute phase reaction to an inciting antigen/pathogen. By comparison, an anti-inflammatory (T_h_2) adaptive immune response serves as a countermeasure to limit T_h_1 responses and thereby facilitate recovery. Exercise may suppress pro-inflammatory immune responses by enhancing anti-inflammatory adaptive immunity (McFarlin et al., 2006). However, findings from this study do not support a measurable exercise-inducible shift in T_h_1:T_h_2 cell bias.

Alternatively, exercise preconditioning may elicit a sustainable protective effect by altering immune function through the activation of the sympathetic nervous system (Bellinger et al., 2008). In response to elevated levels of catecholamines, acute stressors such as exercise produce a profound, albeit transient, lymphocytosis (Martina et al., 1990). Mobilization of T lymphocytes from their sites of lymphopoiesis, traversing the blood stream, with subsequent trafficking into and egress from secondary lymphoid organs is well defined and utilizes a range of ligands and receptors (Springer, 1994; von Andrian and Mackay, 2000). In this study, exercise preconditioning uniquely elicited a sustained increase in total leukocyte retention within popliteal lymph nodes. Exercise-retained nodal leukocytes harvested at onset of disease responded similarly to sedentary controls, proliferating in response to P2-antigen stimulation and releasing measurable quantities of both pro- and anti-inflammatory cytokines. At onset of EAN, nodal leukocytes from exercise-preconditioned rats responded to P2-antigen stimulation by markedly enhancing IL-10 release. These findings, in isolation, are consistent with an exercise-inducible protective shift toward an anti-inflammatory (T_h_2) adaptive immune response. Exercise preconditioning, however, did not alter P2-antigen-stimulated release of IL-6, TNF-α, or IFN-γ from nodal leukocytes harvested at disease onset, suggesting that autoreactive T_h_1 responsiveness was preserved. Not surprisingly, P2-responsiveness of nodal leukocytes harvested from sedentary rats at peak of disease was negligible and consistent with anergy-associated recovery. Unexpectedly, however, nodal leukocytes harvested at peak of disease from exercise-preconditioned rats retained their ability to respond to P2 antigen stimulation. Exercise preconditioning appears to have spared these autoreactive leukocytes from undergoing anergic silencing.

Although a measurable exercise-inducible shift in T_h_1:T_h_2 cell bias is unlikely, a modest increase in the percentage distribution of retained T helper (T_h_; CD4^+^) lymphocytes could be appreciated at EAN onset. However, this change was not statistically significant. The average (pooled) percentage distribution of leukocytes retained in popliteal lymph nodes from both sedentary and forced-exercise conditioned EAN rats consisted of 57.3 ± 2.1% CD4^+^ lymphocytes and 20.2 ± 1.2% CD8^+^ (cytotoxic) lymphocytes. Subset analysis of retained CD4^+^ lymphocytes harvested at disease onset from sedentary EAN rats, however, revealed a statistically significant increase in the percentage of pro-inflammatory CD4^+^CD212^+^ (T_h_1) lymphocytes. Qualitatively similar increases in the percentage distribution of T_h_1 lymphocytes were observed in the blood of sedentary EAN rats. By comparison, preconditioning with forced exercise limited EAN-induced increases in nodal and circulating T_h_1 lymphocytes without altering the percentage distribution of retained or circulating T_h_2 lymphocytes or activated cytotoxic (T_c_) lymphocytes. While these findings are consistent with EAN being predominantly a T_h_1 cell-mediated autoimmune peripheral nerve disorder, the pathogenesis of Guillain-Barré Syndrome (GBS) most likely involves an imbalance between pro-inflammatory (T_h_1, T_h_17) and anti-inflammatory (T_h_2, T regulatory) subsets of CD4^+^ T cells (Zhang et al., 2013). In our hands, the percentage distribution of T_h_17 CD4^+^ lymphocytes retained in popliteal lymph nodes of EAN rats was negligible (data not shown). Given previous findings showing a selective role of regulatory T lymphocytes in EAN recovery (Zhang et al., 2009), the distribution of Tregs at EAN onset and peak was not evaluated in this study.

While the molecular basis for these observations remains unclear, exercise-induced activation of sympathetic noradrenergic responses may alter nodal egress of effector T-cells (Novotny and Kliche, 1986). More recently, sphingosine-1-phosphate (S1P) receptors have emerged as key regulators of lymphocyte egress. S1P receptor activation elicits marked immunosuppression by sequestering effector T lymphocytes within draining lymph nodes (Rosen et al., 2003). *In vivo* application of the prodrug FTY720, a S1P analog, attenuates the development and severity of EAN (Papadopoulos et al., 2010; Galicia-Rosas et al., 2012). Moreover, FTY720 was reported to reduce the nodal content of Foxp3^+^ lymphocytes while increasing the appearance of circulating Foxp3^+^ lymphocytes in rats recovering from EAN (Zhang et al., 2009). Interestingly, physical exercise is reported to increase plasma levels of S1P (Baranowski et al., 2011; Banitalebi et al., 2013). It remains possible that forced exercise preconditioning elicits protection by enhancing S1P-mediated mobilization of regulatory T cells. Further studies are required to determine whether exercise preconditioning limits egress of functionally competent effector T cells while mobilizing regulator T cells from draining lymph nodes in response to elevated plasma levels of S1P.

Here, we report that preconditioning with forced exercise elicits a sustained attenuation of EAN severity, in part, by attenuating egress of functionally competent autoreactive pro-inflammatory lymphocytes without significantly altering T_h_1:T_h_2 cell bias. An unexpected observation was the ability of exercise preconditioning to delay or prevent silencing of retained autoreactive leukocytes. While exercise-induced immunosuppression may represent a sustainable adjunctive therapeutic intervention for the management of inflammatory disorders, additional experimental and phase studies are required to elucidate mechanistic causality and clinical efficacy.
